# Teledermatology Adaptations in the COVID-19 Era

**DOI:** 10.3389/fmed.2021.675383

**Published:** 2021-05-26

**Authors:** Harrison A. Edwards, Xiaohua Shen, H. Peter Soyer

**Affiliations:** ^1^Princess Alexandra Hospital, Woolloongabba, QLD, Australia; ^2^School of Public Health, University of Queensland, Herston, QLD, Australia; ^3^Dermatology Research Centre, The University of Queensland Diamantina Institute, The University of Queensland, Brisbane, QLD, Australia

**Keywords:** teledermatology, telemedicine, COVID-19 pandemic, infection control, clinical epidemiology, health services research [MeSH], clinical pathways, performance and evaluation

## Abstract

The COVID-19 pandemic has required health services worldwide to adapt to dramatically changing healthcare needs and risks across all medical specialties. In the dermatology department at Princess Alexandra Hospital, Brisbane, Australia, we developed and implemented a teledermatology system with 1 week's notice to help reduce infection risk bidirectionally, while saving patients many hours of travel and waiting time with acceptable technological substitutes for the clinical encounters. In this study, we report the efficacy and tolerability of our telephone consultation and store and forward imaging system, including patient experience from validated survey data. Our design, implementation and usage of a remote-default system provides experience and lessons to draw upon in developing future telemedicine systems to address dermatology service maldistribution – an issue affecting large areas of Australia – as well as preparedness for future infection mitigation requirements.

## Introduction

The COVID-19 pandemic has required health services worldwide to adapt to dramatically changing healthcare needs and risks across all medical specialties.

In the dermatology department at Princess Alexandra Hospital, Brisbane, Australia we developed, and instituted a teledermatology system with 1 week's notice. This system comprised of appointments conducted by telephone, and the capacity to receive images by email. Here, we discuss the successes and lessons of implementing this simple system which helped reduce in-person patient numbers and infection risk bidirectionally, while saving patients many hours of travel and waiting time.

Our department has experience in managing a live interactive telehealth dermatology service to treat patients in remote locations (1), as well as a same-day store and forward emergency teledermatology service (2, 3). However, until the current pandemic, face-to-face assessment had been the modality for nearly all routine and urgent appointments and thus comprised the vast majority of patient care.

When the COVID-19 pandemic struck in March 2020 in Brisbane, Australia, and strict lockdown measures were enacted, our dermatology department faced the decision between closing entirely and leaving our catchment of over a million people without a dermatology service, or quickly developing a way that we could continue to serve our patients.

The pre-existing videoconferencing arrangement is limited in that it takes place in the hospital's telehealth center and can only be allocated limited space and time (one session per week), as it uses dedicated hardware and requires a clinic-to-clinic format. Video calls using smartphones would have required either the doctors to use their personal mobile phones and phone numbers, or for the department to acquire and setup additional hardware. These options unfortunately were not feasible, especially in the short time frame and in the context of institutional IT rules.

This study reports on the clinical efficacy and patient experience of a new remote consultation system we devised.

## Method

### Clinic Process

The system devised consisted of administrative staff checking in the patients by phone and confirming their phone number and email address in the hour prior to their booked appointment, which saves doctors time. Additionally, because most patients are waiting comfortably at home rather than in the clinic waiting room, they are less inconvenienced by waiting to be seen.

Patient files are queued by nursing staff, and the medical staff call the patients by phone. An email address with shared staff access was approved and created to receive clinical photos from patients to support the phone consultations when indicated. The images were then incorporated into the usual electronic medical record, Millennium (Cerner Corporation; North Kansas City, Missouri, United States).

**Figure 1:**
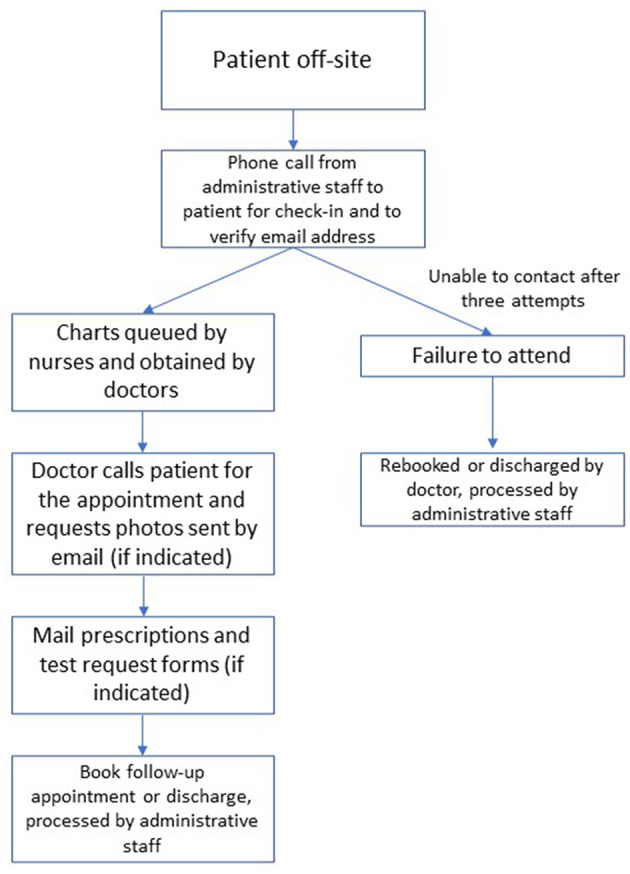
Clinic workflow in the teledermatology system.

Prescriptions and investigation forms are physically mailed to the patient's home address, and urgent prescriptions are faxed directly to the patient's pharmacy for rapid preparation and dispensing (Figure 1).

### System Implementation

This system was developed by nursing and administrative staff in the department with input and feedback from the doctors, and was instituted on 23rd March 2020 after a week of preparation.

At first, dermatology trainees were tasked with determining which upcoming appointments would be suitable for remote consultation. Factors evaluated included patient location, diagnosis, and current management if the appointment was for a review, and expected risk of deterioration and ability to evaluate without in-person examination.

Subsequently, we changed to a system whereby phone consultation was the default. If issues arose during the consultation that would require in-person review (such as an inability to adequately examine the patient using photos, a requirement for discussion of complex management decisions, or urgent physical treatments), a subsequent in-person appointment was made. Patients located nearby could sometimes attend the clinic in-person the same day; for other cases, an appointment would be arranged within a week.

This system of teledermatology by default and escalation when required increased the numbers of consultations conducted remotely and eliminated the time requirement of dermatology trainees assessing appropriateness for teledermatology prior to the appointment.

As the year progressed and government lockdown rules were reduced, more appointments were conducted in-person. In June to September, review appointments could be booked as in-person or teledermatology depending on doctors' clinical judgment and patient preference, whereas appointments for new patients were booked for teledermatology. During this period, appointments conducted in-person approached 50%.

By November, in-person appointments became the default, and subsequently ~20% of appointments were conducted by teledermatology.

### Evaluation

To assess the implementation of this system, clinic appointment counts were assessed and compared with the same period in the calendar year prior, appointments requiring a language interpreter were noted, and a count of cases in the email system was conducted.

For the patient perspective, a survey was conducted by phone of 100 patients of the ~2,000 patients who had a phone appointment between 23rd March 2020 and 15th January 2021. To minimize recall bias and to elicit the most accurate and detailed responses, this sample of patients was taken from those who had a phone appointment within the recent time frame of 18th November 2020 to 15th January 2021.

A validated survey tool, the Telehealth Usability Questionnaire (TUQ), was used to comprehensively cover the domains of usefulness, ease of use and learnability, interface quality, interaction quality, reliability, satisfaction and future use. The TUQ has demonstrated strong validity and reliability across various telecommunication systems, whereas most other questionnaires apply primarily to special purpose videoconferencing hardware or other single modalities (4).

The TUQ was modified to exclude questions that were not applicable to our context. Consequently, a set of 17 questions (based on a 7-point Likert scale) and an open-ended feedback question were used.

## Results

Through this system, in-person clinic attendance was reduced by 95% at the initial peak of the pandemic while maintaining high clinical throughput: a sample of medical dermatology clinic sessions in the first 2 months of the teledermatology system showed higher patient numbers than the same period the year prior.

However, surgical clinic sessions were more negatively impacted by the pandemic: although appointment counts under the teledermatology system were only 11% lower than usual, these appointments served only as a safety check – urgent, complex lesions were subsequently booked for in-person management; patients were otherwise advised to see their general practitioner for other skin concerns.

A language interpreter was required in 5% of cases, and surprisingly, an evaluation of the email system in the first 3 weeks demonstrated that photos were required in only 36.5% (152/417) of appointments. When photos were required, patients could usually manage to send them by email to the shared inbox described above, and photos were generally of suitable quality. The proportion of consultations over the same period that required escalation to an in-person encounter was low, at 2.4% (10/417).

**Figure 2:**
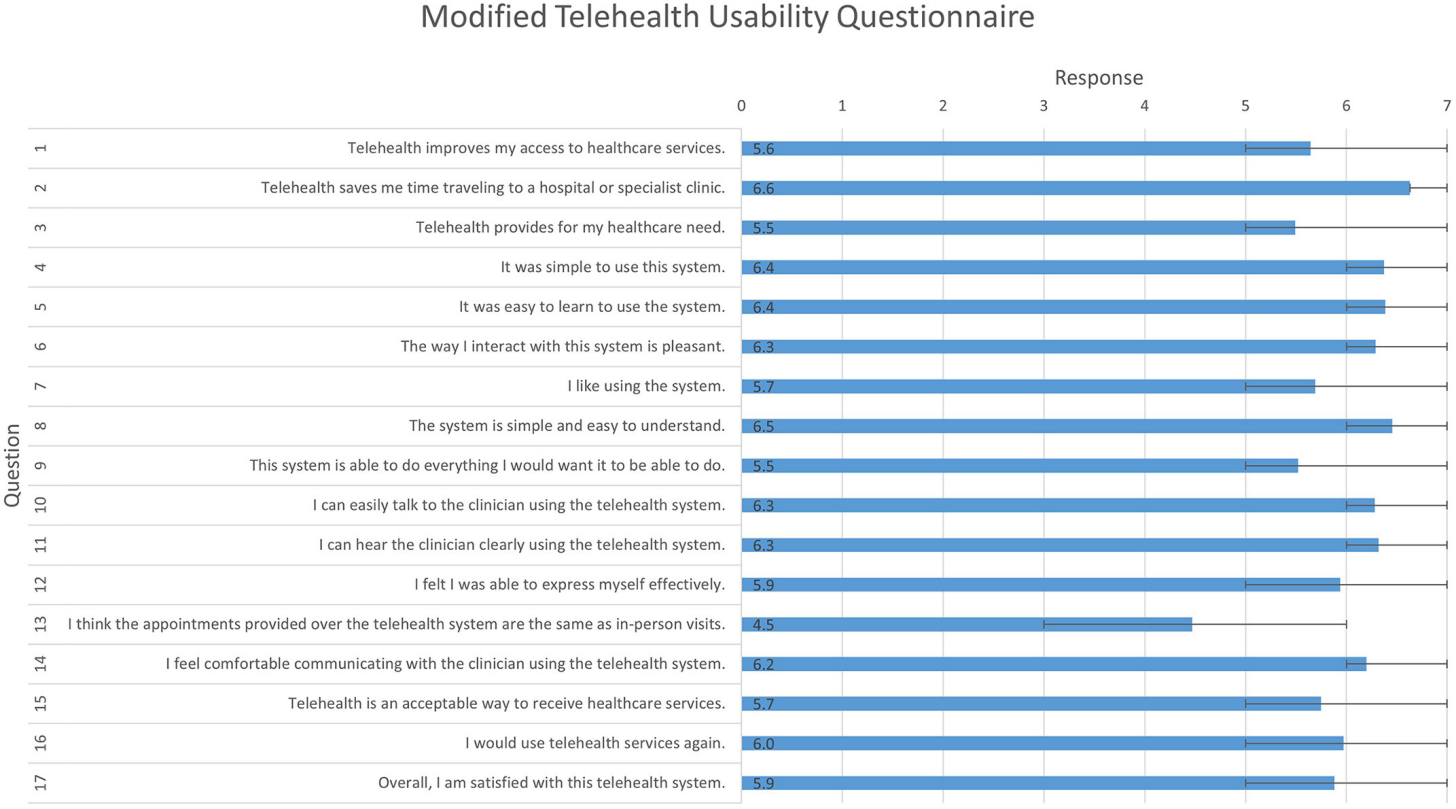
Modified Telehealth Usability Questionnaire, responses to quantitative questions. Likert scale; 1 (disagree) to 7 (agree); mean and interquartile range.

The major benefits identified from the survey (Figure 2) were saving travel time (Q2) and that it is simple and easy to understand and use (Q4, Q5, Q8).

Comparatively low scores were given for questions that asked about clinical equivalence to in-person appointments (Q3, Q9, Q13). However, most patients responded indicating that they were satisfied with the service and that they would use it again (Q16, Q17).

Based on 74 responses to the open-ended feedback question, certain benefits and drawbacks were identified. Some patients (5%), especially the elderly, identified difficulty with taking and emailing photos. Some patients (3%) whose phone appointment was not clinically adequate and required a subsequent in-person appointment were unhappy about the double handling and delay.

Many patients (20%) emphasized the benefit of eliminating travel time and reducing the interruption to their day, but many (27%) also felt that dermatology was particularly challenging for telemedicine due to the primacy of visual examination. Relatedly, a contingent of patients (7%), including some of those who reported overall satisfaction with the teledermatology system, stated a desire for periodic in-person appointments.

Despite the reduction in travel time, several patients (9%) expressed inconvenience regarding the unreliable appointment wait times on the day of their appointment. A surprisingly small number of patients (4%) commented on infection risk mitigation as a benefit of the teledermatology system.

## Discussion

### Strengths

Our simple conventional phone-based teledermatology system has worked well, allowing patients to still receive clinical care while avoiding bidirectional infection risk. Although existing literature on teledermatology essentially always involves remote visual examination (either synchronously or asynchronously) (5, 6), our finding that images were required only in a minority of cases suggests that a large proportion of teledermatology, more feasibly follow-up appointments, could be conducted without visual examination. This may be due to the department's new:review ratio of 1:4; however, image requirement by appointment type could not be extracted to confirm this.

Administrative workload has been mostly unchanged in the new, remote system, and time-consuming steps such as patient and staff movement between waiting rooms and consultation rooms have been avoided.

This system is advantageous for reducing infection risk for the general patient population, but is especially protective for our many patients who are on immunosuppressive therapies. As well as reducing bidirectional infection risk, teledermatology reduces the need for personal protective equipment, helping to maintain supplies for clinical encounters where it is necessary.

As a department of a public hospital, we have the advantage that we do not rely on private health insurance or Medicare billing, and therefore issues related to those aspects can be avoided in our teledermatology process.

### Weaknesses

Many patients were able to send clinical photos through the email system, but as described above, 33% of survey respondents either found the email system technically challenging (5%) or felt it was inferior compared to in-person visual examination (27%). Email was used to receive photos from patients, even though it is not an institutionally approved way of communicating with patients outside of emergency conditions such as the COVID-19 pandemic. The other store and forward methods requested by patients, Facebook Messenger and Multimedia Messaging Service (MMS), would have carried additional issues of privacy and difficulty of shared staff access.

Evaluation of patients for skin cancer is problematic in this teledermatology system, because of the difficulty of replicating examination electronically and the significant consequences of incorrect skin cancer diagnosis. Each lesion on a patient poses new diagnostic uncertainty, and biopsies cannot be done remotely. Consequently, skin cancer patients requiring urgent biopsy comprised many of the patients needing to be seen in-person. Generally, patients were advised that their skin cancer examination would be delayed and that they should see their GP in the meantime.

Appointments requiring a language interpreter were sometimes complicated. These were arranged either as a consultation with an interpreter co-located with the doctor and the patient on the phone, or as a three-way phone conversation with the doctor in the clinic and the interpreter and patient on the phone. If the dermatology consultant needed to be involved, the three-way call between the doctor, patient and interpreter would need to be ended and later reconnected to relay the consultant's input, which was cumbersome and time consuming.

## Conclusion

This system was put in place after only 1 week of development, reduced the number of patients in the clinic by 95%, allowing us to greatly reduce the infection risk for both patients and staff, reduce PPE requirement, and also save patients travel time and waiting time.

The necessity of telemedicine systems to minimize infection risk during the current pandemic has been recognized in Australia and overseas. Health systems worldwide have responded by declaring that in-person medical care should be limited to only the most urgent patients. To facilitate this, they have reduced administrative and security requirements for telemedicine, allowing healthcare providers to serve patients through everyday communications technologies such as phone, email, instant messaging, and video chat software (7, 8).

Dermatology departments in the United States (9–11), China (12), and Europe (13–15) have accordingly restricted their in-person clinical encounters due to the recognized risk of SARS-CoV-2 transmission. Similarly, the Australian government has adapted administrative billing requirements for telemedicine by general practitioners and all medical specialists (16).

Our experience of urgently designing and implementing a teledermatology system was broadly positive, due to the benefits of infection risk reduction, reduced patient travel and waiting times, and acceptability to patients and doctors of in-person history and examination substitutes.

In the United States, The Society of Dermatology Hospitalists developed an algorithm to triage teledermatology consults. A high rate of teledermatology suitability was found, with only four out of the 35 patients sampled (11%) requiring escalation to in-person consultation (17), which accords with our result of 2.4%.

As before the COVID-19 pandemic, there is legitimate concern regarding technological security and patient privacy regarding teledermatology. Our imperfect but successful design, implementation and usage of a remote-default system using phone for consultations with supporting images sent by email provides experience and lessons to draw upon in developing future robust telemedicine systems to address dermatology service provision, taking into consideration specialist maldistribution in Australia as well as preparedness for future infection mitigation requirements.

## Data Availability Statement

The raw data supporting the conclusions of this article will be made available by the authors, without undue reservation.

## Ethics Statement

The studies involving human participants were reviewed and approved by Metro South Health Human Research Ethics Committee. Written informed consent for participation was not required for this study in accordance with the national legislation and the institutional requirements.

## Author Contributions

HE and XS contributed to data acquisition. HE and HS contributed to data analysis. All authors contributed equally to the concept, design, and contributed to the drafting and critical revision of the manuscript.

## Conflict of Interest

HS is a shareholder of MoleMap NZ Limited and e-derm consult GmbH, undertakes regular teledermatological reporting for both companies, and Medical Consultant for Canfield Scientific Inc., Revenio Research Oy and also a Medical Advisor for First Derm. The remaining authors declare that the research was conducted in the absence of any commercial or financial relationships that could be construed as a potential conflict of interest.
